# Antifungal Activity of Eugenol Analogues. Influence of Different Substituents and Studies on Mechanism of Action

**DOI:** 10.3390/molecules17011002

**Published:** 2012-01-19

**Authors:** Héctor Carrasco, Marcela Raimondi, Laura Svetaz, Melina Di Liberto, María V. Rodriguez, Luis Espinoza, Alejandro Madrid, Susana Zacchino

**Affiliations:** 1 Departamento de Ciencias Químicas, Universidad Andrés Bello, Campus Viña del Mar, Los Fresnos N° 52, Viña del Mar 2520000, Chile; 2 Área Farmacognosia, Facultad de Ciencias Bioquímicas y Farmacéuticas, Universidad Nacional de Rosario, Suipacha 531, 2000-Rosario, Argentina; Email: mpraimondi@hotmail.com (M.R.); 3 Departamento de Microbiología, Facultad de Ciencias Médicas, Universidad Nacional de Rosario, Santa Fe 3100, 2000-Rosario, Argentina; 4 Área Biología Vegetal, Facultad de Ciencias Bioquímicas y Farmacéuticas, Universidad Nacional de Rosario, Suipacha 531, 2000-Rosario, Argentina; 5 Departamento de Química, Universidad Técnica Federico Santa María, Av. España N° 1680, Valparaíso 2340000, Chile; Email: luis.espinozac@usm.cl (L.E.)

**Keywords:** eugenol derivatives, antifungal activity, mechanism of antifungal action, lipophilicity, SAR

## Abstract

Twenty one phenylpropanoids (including eugenol and safrole) and synthetic analogues, thirteen of them new compounds, were evaluated for antifungal properties, first with non-targeted assays against a panel of human opportunistic pathogenic fungi. Some structure-activity relationships could be observed, mainly related to the influence of an allyl substituent at C-4, an OH group at C-1 and an OCH_3_ at C-2 or the presence of one or two NO_2_ groups in different positions of the benzene ring. All active compounds were tested in a second panel of clinical isolates of *C. albicans* and non-*albicans Candida* spp., *Cryptococcus neoformans* and dermatophytes. The eugenol derivative 4-allyl-2-methoxy-5-nitrophenol (**2**) was the most active structure against all strains tested, and therefore it was submitted to targeted assays. These studies showed that the antifungal activity of **2** was not reversed in the presence of an osmotic support such as sorbitol, suggesting that it does not act by inhibiting the fungal cell wall synthesis or assembly. On the other hand, the Ergosterol Assay showed that **2** did not bind to the main sterol of the fungal membrane up to 250 µg mL^−1^. In contrast, a 22% of fungal membrane damage was observed at concentrations = 1 × MIC and 71% at 4× MIC, when **2** was tested in the Cellular Leakage assay. The comparison of *log P* and MICs for all compounds revealed that the antifungal activity of the eugenol analogues would not to be related to lipophilicity.

## 1. Introduction

Fungi have emerged over the past two decades as major causes of human infections, especially among immunocompromised hosts, having an enormous impact on morbidity and mortality [1,2]. A matter of concern in the treatment of fungal infections is the limited number of efficacious antifungal drugs which are not completely effective for the eradication of mycoses [3,4]. In addition, they all possess a certain degree of toxicity and develop quickly resistance due to the large-scale use [5]. There is, therefore, an urgent need for new antifungal chemical structures as alternatives to the existing ones [6].

Some studies on the antifungal activity of eugenol (**1**) [the main constituent of the essential oils of *Pimenta racemosa* (bay leaves), *Cinnamomum verum* (cinnamon leaf) and *Syzygium aromaticum* (clove)] and analogues, have led to contradictory results. Zemek *et al.* [7] reported that **1** (possessing a 4-allyl group) was almost inactive (MICs = 3,000 µg mL^−1^) against *Saccharomyces cerevisiae*, *Candida* *albicans* and *Aspergillus niger* while isoeugenol **20** [which possesses a 4-(2′-propenyl) substituent] exhibited a moderate inhibitory effect on the same fungi with MICs 100–250 µg mL^−1^ in broth dilution methods.

On the other hand, Kubo *et al.* [8] reported that both **1** and safrole (**12**) (with a 3,4-methylenedioxy-2′-propenyl substituent) possess moderate activity against *S. cerevisiae*, *Candida utilis*, *Pityrosporum ovale*, and *Penicillum chrysogenum*, with MICs between 100 to 800 µg mL^−1^ in broth dilution methods with shaking, being *P. ovale* the most sensitive fungus. In a second report, Kubo *et al.* [9] reported that **1** and **12** possessed moderate activity against *C. albicans* (MICs = 800 and 200 µg mL^−1^ respectively) with shaking. In the third paper of this series, Kubo *et al*. [10] reported that **12** was active against *S. cerevisiae* at 200 µg mL^−1^ without shaking. This paper also suggests that both the propenyl and the allyl moieties appeared to be the minimum requirements for these phenylpropanoids to show antifungal activity. 

Meanwhile, we have reported the antifungal properties in agar dilution assays of a series of phenylpropanoids against yeasts, *Aspergillus* spp. and dermatophytes [11], finding that **1** and some of its analogues were inactive on all fungal spp. up to 50 µg mL^−1^.

In addition, Faria *et al.* [12] reported that **1** displayed antifungal activity against the phytopathogenic fungi *Alternaria* sp. and *P. chrysogenum* but it was inactive against *A. niger*, *Botryosphaeria rhodina* or *Rhizoctonia* sp. in agar diffusion assays.

In turn, Wang *et al.* reported that **1** possessed antifungal activity inhibiting the wood decay fungi *Coriolus versicolor* and *Laetiporus sulphureus* [13], in agar dilution assays at a single concentration of 100 µg mL^−1^.

In a more recent paper, Campaniello *et al.* [14] found that **1** at concentrations = 100–150 µg mL^−1^ is an effective antifungal compound against phytopathogenic *Aspergillus*, *Penicillium*, *Emericella* and *Fusarium* spp., suggesting that this activity could be attributed, in part, to the presence of a phenolic group.

Unfortunately, these important antifungal studies were performed with non-standardized either qualitative or quantitative tests which prevent the comparison of results. In a recent paper, Cos *et al.* [15] stated that the use of a primary standardized validated primary screening assay is essential to guarantee confident and reproducible results. In this regard, the Clinical and Laboratory Standards Institute (CLSI), formerly National Committee for Clinical and Laboratory Standards (NCCLS) established consensus’ procedures to facilitate the agreement among laboratories in measuring the susceptibility of yeasts (document M-27 A2 [16], updated in 2008 as M-27 A3 [17]) and of filamentous fungi (document M-38 A [16], updated in 2008 as M-38 A2 [17]) to antifungal agents, with broth dilution methods. The standardized parameters detailed in both documents included preparation of antifungal stock solutions, dilutions for testing, inoculum preparation, inoculum size, choice among several synthetic media, temperature and duration of incubation, endpoint definitions and reference MIC ranges for microdilution testing of both, the established and newly introduced antifungal agents.

Regarding studies on the mechanism of action of eugenol and analogues, Chami *et al.* suggested [18] that the anticandidal action of **1** could be attributed to the damage of the envelope of fungal cells. Unfortunately, this work did not discriminate the target between membrane or cell-wall. 

In parallel, Sikemma *et al.* [19] and Gill *et al.* [20] found that the antibacterial mechanism of action of eugenol is the disruption of the cytoplasmic membrane, which could be due to the fact that the phenolic hydroxyl group might increase the solubility of this molecule in aqueous suspensions improving the ability to pass through the hydrophilic portion of the cell envelope. This assertion is in clear contradiction to a QSAR study of essential oils’ components performed by Voda *et al.* [21], who found that the best antifungal activities were displayed by the most hydrophobic phenylpropanoids which possess a higher ability to penetrate the walls of fungal cells than the hydrophilic ones. 

Considering the dissimilar results reported on the antifungal activity of **1** and analogues described above, a more systematic investigation of the antifungal activities of phenylpropanoids comprising: (i) a large number of compounds; (ii) utilizing CLSI methodologies; (iii) using the same fungal panel; seems in order, to arrive at confident and comparable results. In addition, some targeted assays on the most active structures were used to discriminate whether active compounds damage either the membrane or the wall of the fungal cells and to add new data on the mechanism of antifungal action of this type of compounds.

## 2. Results and Discussion

Phenylpropanoids **1–21**, differing in the pattern of substitution on the benzene ring, were evaluated for antifungal properties with standardized non-targeted as well as targeted assays, with the aim of determining the role of the different substituents in the antifungal behavior and to obtain some evidence about their mechanism of action.

**Figure 1:**
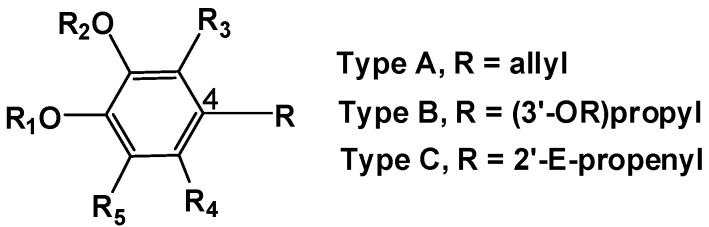
Analogues of eugenol grouped according to the 4-substituent.

For the sake of clarity, all compounds were grouped in three types [A (**1**–**13**); B (**14**–**19**); C (**20**–**21**)] according to their 4-substituent (Figure 1).

### 2.1. Chemistry

**Scheme 1:**
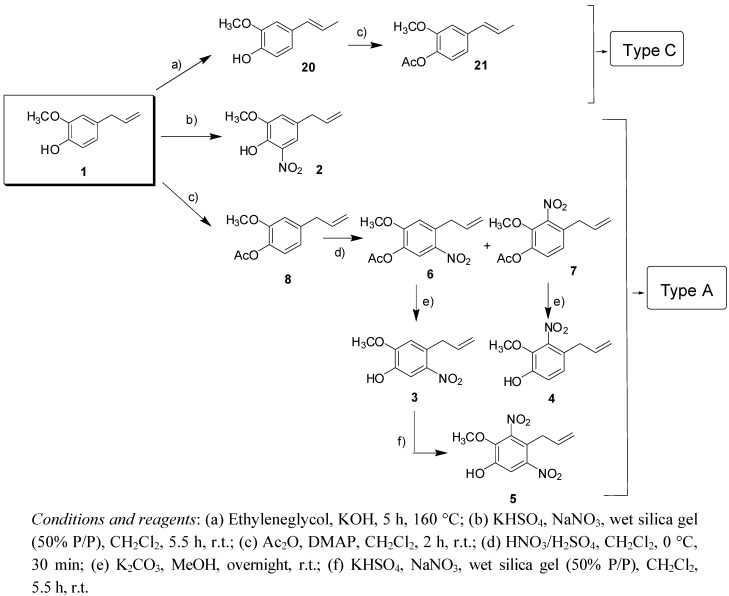
General synthesis scheme of derivatives of eugenol.

From natural eugenol (**1**) [22] both, the type A allyl-compounds **2**–**8** and the type C isopropenyl derivatives **20** and **21** were obtained by typical acetylation, isomerization and nitration procedures (Scheme 1).

**Scheme 2:**
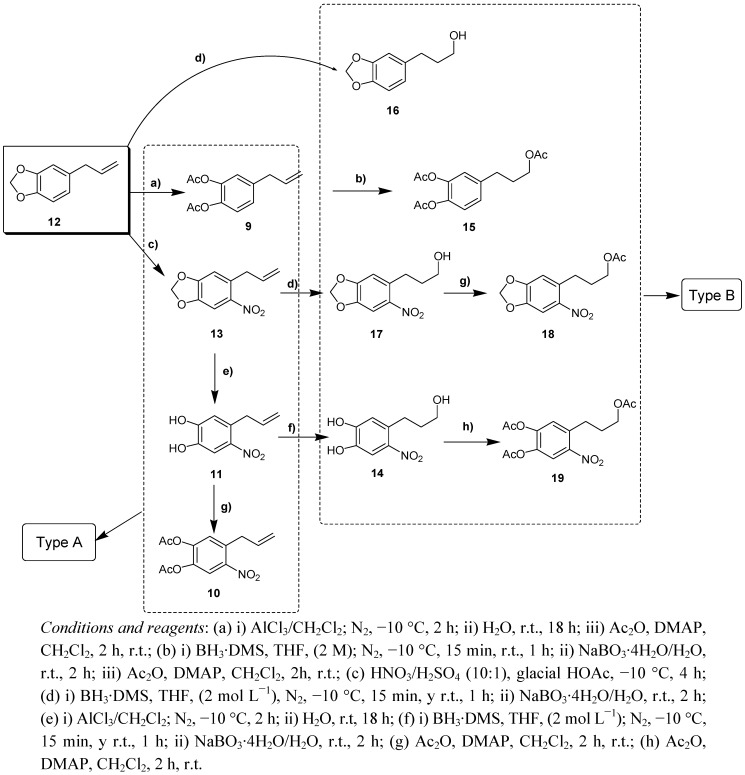
General synthesis scheme of derivatives of safrol.

On the other hand, from commercial safrole (**12**) both the type A derivatives **9**,**10**,**11**,**13** as well as the type B-propyl analogues **14**–**19** were obtained with the following reactions: opening the methylenedioxy group with AlCl_3_/CH_2_Cl_2_, treatment of the allyl group with borane under a nitrogen atmosphere (and subsequent acetylation to afford the 3′-OAc propyl group) and/or nitration with the appropriate reagents (Scheme 2).

Compounds **1**, **2**, **6**–**8**, **20** and **21** are known structures [23,24], while **3**–**5**, **9**–**11**, **13**–**19** were new compounds. Their structures, which were consistent with the proposed structures, were assigned by ^1^H- and ^13^C-NMR and mass spectroscopy (see Experimental).

### 2.2. Antifungal Activity

Minimum Inhibitory concentrations (MIC) of compounds **1**–**21** were determined against a panel of fungal strains with the microbroth dilution method following the CLSI guidelines, which constitutes a first order evaluation. Then, the most active compounds were submitted to second order studies consisting in both the testing of them against a second panel of clinical isolates and the evaluation of the most active compounds with targeted assays to obtain some evidence of their mode of action.

#### 2.2.1. First Order Studies

To carry out the antifungal evaluation, concentrations of compounds up to 250 µg mL^−1^ were incorporated to growth media according to published procedures [27,28]. Amphotericin B, terbinafine, and ketoconazole were used as positive controls. Table 1 summarizes the concentration of compounds that completely inhibited the growth (MIC_100_) of nine opportunistic pathogenic fungi including yeasts (*C. albicans*, *Cryptococcus neoformans*, *S. cerevisiae*), as well as dermatophytes (*Microsporum* and *Trichophyton* spp.). None of them inhibited *Aspergillus* spp.

Although the activity displayed by all compounds was moderate, it is interesting to note some apparent structure-activity relationships that might be useful for the future design of analogues with better antifungal behavior.

*(a) Influence of substituents on C-4*: the results of Table 1 suggest that the 4-allyl moiety plays a positive role in the antifungal behavior of this series, since all type A-compounds possessing this group (compounds **1**–**13**) display antifungal activities (MICs < 250 µg mL^−1^) against at least one fungus. In contrast, compounds **14**–**21**, which do not possess it, are almost inactive. To better understand the role of the allyl radical in the antifungal properties of this series, we compared the activity of seven pairs of compounds (**1**/**20**; **8**/**21**; **9**/**15**; **10**/**19**; **11**/**14**; and **13**/**17**). This change resulted in the disappearance of the antifungal activity.

*b) Role of the OH in C-1*: The comparison of the activities of the pair of compounds **1**/**8**; **3**/**6**; **4**/**7**; and **20**/**21** the first of each pair-component with a free phenolic OH and the second with an acetate esterifying it, showed that the phenolic OH did not have any influence on the activity since similar activities were observed for both components of each pair. Instead, the comparison of activities of pairs **1**/**12** and **3**/**13** in which the substitution pattern (1-OH, 2-OMe) was replaced by (1,2-OCH_2_O-) showed a decrease in the antifungal properties. Both results are in contrast with those previously reported [21], which suggested that the antifungal activity of eugenol could be attributed to the presence of a phenolic group that would form H-bonds with active sites of target enzymes.

*(c) Role of the OCH_3_ in C-2*: Two of the six pairs of compounds included in the preceeding section (**1**/**12** and **3**/**13**) must be analyzed again, this time from the point of view of the presence of 2-OCH_3_. As stated above, it is observed a clear decrease in the antifungal properties when the OCH_3_ is changed to another group. In fact, **12** is completely devoid of activity while **1** is active against four strains with MICs of 125–250 µg mL^−1^. In turn, **3** possesses a broader spectrum of action (six strains) than **13** (two strains), although the MICs are similar for both compounds against the sensitive strains. The other comparable pairs of compounds **3**/**11**, **6**/**10**, and **8**/**9** in which a 2-OCH_3_ was replaced by a 2-OH (**3**/**11**) or a 2-OAc (**6**/**10** and **8**/**9**), did not show differences in the antifungal activity.

*(d) Influence of NO_2_ groups in positions 3, 5 and 6 of the benzene ring*: The introduction of a NO_2_ group on different positions (3, 5 and 6) of **1** (**1**→ **4**, **1**→**3** and **1**→**2** respectively) led to an increase of the antifungal activities when analyzed from both the point of view of the broadening of the spectrum of action and decreased MICs.

**Table 1:** MIC values (µg mL^−1^) of eugenol (**1**) and analogues **2**–**21** against human opportunistic pathogenic fungi. 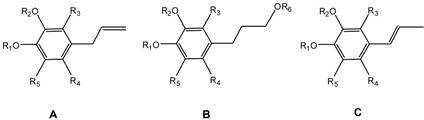

	Type	R_1_	R_2_	R_3_	R_4_	R_5_	R_6_	*Log P*	*Ca*	*Sc*	*Cn*	*Tr*	*Tm*
**1**	A	H	CH_3_	H	H	H	-	2.57	i	i	250	125	125
**2**	A	H	CH_3_	H	H	NO_2_	-	2.65	31	62	16	31	31
**3**	A	H	CH_3_	H	NO_2_	H	-	2.65	250	250	125	62	62
**4**	A	H	CH_3_	NO_2_	H	H	-	2.65	250	125	125	31	31
**5**	A	H	CH_3_	NO_2_	NO_2_	H	-	2.61	i	i	i	250	250
**6**	A	Ac	CH_3_	H	NO_2_	H	-	2.77	125	i	250	62	62
**7**	A	Ac	CH_3_	NO_2_	H	H	-	2.77	250	i	250	62	62
**8**	A	Ac	CH_3_	H	H	H	-	2.55	i	i	i	125	125
**9**	A	Ac	Ac	H	H	H	-	2.26	250	i	250	125	125
**10**	A	Ac	Ac	H	NO_2_	H	-	2.38	250	i	125	125	125
**11**	A	H	H	H	NO_2_	H	-	2.13	250	i	125	62	62
**12**	A	-CH_2_-		H	H	H	-	2.87	i	i	i	i	i
**13**	A	-CH_2_-		H	NO_2_	H	-	2.14	125	i	250	i	i
**14**	B	H	H	H	NO_2_	H	H	1.21	i	i	i	i	i
**15**	B	Ac	Ac	H	H	H	Ac	1.56	i	i	i	i	i
**16**	B	H	H	H	H	H	H	1.94	i	i	i	i	i
**17**	B	-CH_2_-		H	NO_2_	H	-	1.22	i	i	i	i	i
**18**	B	H	H	H	NO_2_	H	Ac	1.81	250	i	250	125	125
**19**	B	Ac	Ac	H	NO_2_	H	Ac	2.05	i	i	i	i	i
**20**	C	H	CH_3_	H	H	H		2.52	i	i	i	i	i
**21**	C	Ac	CH_3_	H	H	H	-	2.50	i	i	i	i	i
Amphotericin B									0.78	0.50	0.25	0.075	0.075
Terbinafine									1.56	3.12	0.39	0.01	0.025
Ketoconazole									0.50	0.50	0.25	0.025	0.025

i = inactive (MIC > 250 μg mL^−^^1^).

Regarding non-phenolic type-A compounds, the introduction of a NO_2_ group on the 5-position of the non-phenolic analogues of **1**, 1,2-diacetate-4-allylbenzene (**9**) and 1,2-methylenedioxy-4-allylbenzene (**12**) produced no changes in activity, *i.e*., compound **10** displays similar activities than **9** and compound **13** is likewise as inactive as **12**.

The comparison of the activities of **2**, **3** and **4** against each other, allows one to have a look into the influence of the NO_2_-position in type A-phenolic compounds, which diminishes in the order 6 (**2**) > 3 (**4**) > 5 (**3**). In contrast, different locations (3 and 5) of the NO_2_ group in the non-phenolic analogues **6** and **7** did not produce any change in the antifungal activity.

The introduction of a second NO_2_ group on compounds **3** or **4** led to 3,5-dinitroeugenol (**5**), which showed a narrower spectrum of action as well as a lower antifungal activity. Added to the results obtained with type A-derivatives, a 5-NO_2_ group on the type B-inactive phenolic compound **15** led to the also inactive compound **19**.

#### 2.2.2. Second Order Studies

*(a) Antifungal activity of active structures on clinical isolates of Candida spp.*: In order to gain insight into the spectrum of activity of eugenol analogues, the three most active compounds against *C. albicans* (phenolic **2**, non-phenolic acetate **6** and methylenedioxy derivative **13**, representative each one of the different type A-derivatives) were tested against an extended panel of clinical isolates of *C. albicans* and non-*albicans Candida* spp. 

MIC values of the three compounds were determined against this new panel by using three endpoints: MIC_100_, MIC_80_ and MIC_50_ (the minimum concentrations of compounds that inhibited 100, 80 and 50% of growth respectively). The application of a less stringent end-point such as MIC_80_ and MIC_50_ has been shown to consistently represent the *in vitro* activity of compounds [16,17] and many times provides a better correlation with the *in vivo* behavior [25,26].

In addition to MIC determinations, the evaluation of MFC of each active compound against this extended panel was accomplished by sub-culturing a sample from MIC tubes showing no growth, onto drug-free agar plates.

The selection of *Candida* strains was due to the importance that this fungal genus possesses in the epidemiology of fungal infections [27]. It is known that *Candida* spp. are among the leading causes of nosocomial blood stream infections worldwide and, although *C. albicans* was in the past the usual *sp.* associated with invasive infections, at present *non-albicans Candida* spp. such as *C. tropicalis*, *C. glabrata*, *C. parapsilopsis*, *C. krusei* and others, comprise more than half of the isolates of candidosis in human beings [27].

Results (Table 2) show that compound **2** possessed very similar MIC_100_, and was fungicide, against all *C. albicans* strains including the standardized one and showed MIC_50_ values <25 µg mL^−1^. In turn, non-*albicans Candida* clinical strains were equally sensitive to **2** than the standardized one and compounds **6** and **13** showed very low MIC_50_ values (4–8 µg mL^−1^ and 15 µg mL^−1^) for two and three strains respectively.

*(b) Antifungal activity of active structures on clinical isolates of Cryptococcus neoformans*: Compounds **2**–**4**, **10** and **11** which displayed the better activities against *C. neoformans* ATCC 32264 were tested against an extended panel of nine clinical isolates of the same fungal sp. and MIC_100_, MIC_80_ and MIC_50_ values for each compound were determined.

The selection of *C. neoformans* was due to the fact that this fungus remains an important life-threatening complication for immunocompromised hosts, particularly for patients who have undergone solid organ transplants and therefore, new compounds acting against this fungus are highly welcome [28,29].

**Table 2:** Minimum Inhibitory Concentrations (MIC_100_, MIC_80_ and MIC_50_) and Minimum Fungicidal Concentration (MFC), in µg mL^−1^ of **2**, **6** and **13** against clinical isolates of *C*. *albicans* and non-*albicans Candida* spp. For the sake of comparison the **MIC** and **MFC** of all compoundsagainst an ATCC standardized strain of *C. albicans* was included.

Strain		2				6				13				Amph. B
	Voucher specimen	MIC_100_	MIC_80_	MIC_50_	MFC	MIC_100_	MIC_80_	MIC_50_	MFC	MIC_100_	MIC_80_	MIC_50_	MFC	MIC_100_
*C. albicans*	ATCC 10231	31	16	8	125	62	62	31	>250	31	16	8	125	1.00
*C. albicans*	C 126-2000	31	25	20	250	i	250	125	>250	31	25	20	250	1.56
*C. albicans*	C 127-2000	62	31	25	125	i	i	i	>250	62	31	25	125	0.78
*C. albicans*	C 128-2000	62	31	16	250	16	16	16	>250	62	31	16	250	1.56
*C. albicans*	C 129-2000	31	25	16	250	i	250	250	>250	31	25	16	250	0.78
*C. albicans*	C 130-2000	62	31	25	250	i	i	i	>250	62	31	25	250	0.39
*C. glabrata*	C 115-2000	125	125	125	250	i	i	i	>250	125	125	125	250	0.39
*C. parapsilopsis*	C 124-2000	125	62	31	>250	i	250	125	>250	125	62	31	>250	0.78
*C. lusitaniae*	C 131-2000	62	50	25	250	i	i	250	>250	62	50	25	250	0.39
*C. colliculosa*	C 122-2000	62	31	25	250	31	31	16	>250	62	31	25	250	0.36
*C. krusei*	C 117-2000	125	100	50	>250	i	i	i	>250	125	100	50	>250	0.39
*C. kefyr*	C 123-2000	125	62	31	>250	i	i	i	>250	125	62	31	>250	0.78
*C. tropicalis*	C 131-1997	62	31	25	>250	i	i	i	>250	62	31	25	>250	0.50

MIC_100_, MIC_80_ and MIC_50_: concentration of a compound that induced 100, 80% or 50% reduction of the growth control respectively. Within Voucher specimen: ATCC = American Type Culture Collection (Rockville, MD, USA); C = Mycological Reference Center (Rosario, Argentina), *C. albicans* = *Candida albicans*; *C. glabrata = Candida glabrata*; *C. parapsilopsis = Candida parapsilopsis*; *C. lusitanae = Candida lusitaniae*; *C. colliculosa = Candida colliculosa*; *C. krusei = Candida krusei*; *C. kefyr = Candida kefyr*; *C. tropicalis = Candida tropicalis*; *C. neoformans = Cryptococcus neoforman*. Amph. B = Amphotericin B.

Results showed (Table 3) that, the activity of each compound against all clinical strains was similar. Nevertheless, it is noteworthy that **2** showed the highest MIC_50_, with values between 4 and 16 µg mL^−1^, which positions this compound as a potential lead for the development of an antifungal drug.

**Table 3:** Minimum Inhibitory Concentrations (MIC_100_, MIC_80_ and MIC_50_) and Minimum Fungicidal Concentration (MFC) of eugenol derivatives **2–4**, **10** and **11** against clinical isolates of *Cryptococcus neoformans*. For the sake of comparison, the **MIC** and **MFC** valuesof both compounds against an ATCC standardized strain of *C. neoformans* are included.

			2				3				4				10				11				Amp. B	Itz
Fungal sp.		Voucher specimen	MIC_100_	MIC_80_	MIC_50_	MFC	MIC_100_	MIC_80_	MIC_50_	MFC	MIC_100_	MIC_80_	MIC_50_	MFC	MIC_100_	MIC_80_	MIC_50_	MFC	MIC_100_	MIC_80_	MIC_50_	MFC	CIM_100_	
*Cn* ATCC 32264			16	8	8	62	125	62	31	250	125	62	31	125	125	62	31	250	125	62	62	>250	0.25	0.15
*Cn*	IM 983040		31	16	8	250	125	62	62	250	125	31	16	125	250	250	125	>250	250	125	16	250	0.13	<0.015
*Cn*	IM 972724		31	16	8	250	125	125	62	250	125	31	31	125	i	i	250	>250	250	125	16	250	0.06	0.25
*Cn*	IM 042074		31	16	8	250	125	125	62	250	125	62	31	125	250	250	31	>250	250	125	62	250	0.25	<0.015
*Cn*	IM 983036		31	16	16	250	125	62	31	250	125	62	62	125	250	250	31	>250	250	125	31	250	0.13	<0.015
*Cn*	IM 00319		31	16	8	250	125	31	31	250	125	62	15	125	250	125	62	>250	250	125	62	250	0.25	<0.015
*Cn*	IM 972751		31	16	8	250	125	62	31	250	125	62	31	250	250	250	62	>250	125	62	62	250	0.25	<0.015
*Cn*	IM 031631		31	16	4	250	250	125	31	250	125	62	31	250	250	250	125	>250	125	125	16	250	0.13	0.25
*Cn*	IM 031706		62	31	16	125	125	62	15	250	125	62	15	250	250	125	31	>250	250	125	31	250	0.25	0.50
*Cn*	IM 961951		31	16	8	250	250	125	62	250	125	62	15	>250	250	125	31	>250	250	62	31	250	0.06	<0.015

MIC_100_, MIC_80_ and MIC_50_: concentration of a compound that induced 100, 80% or 50% reduction of the growth control respectively. Within Voucher specimen: ATCC = American Type Culture Collection (Rockville, MD USA); IM = Malbrán Institute (Buenos Aires, Argentina). *Cn = Cryptococcus neoformans*. Amp. B = Amphotericin B; Itz = Itraconazole.

*(c) Antifungal activity of active structures on clinical isolates of dermatophytes*: Compounds **2–4**, **6**–**11** and **18** which displayed MIC values <125 µg mL^−1^ against dermatophytes of the first panel (see Table 1), were tested against six clinical isolates of each *T. mentagrophytes* and *T. rubrum* (Table 4). The selection of *Trichophyton* spp. was due to the fact they are the cause of 80–93% of chronic and recurrent dermatophyte infections in human beings. They are the ethiological agents of tinea unguium (producer of invasive nail infections), tinea manuum (palmar and interdigital areas of the hand infections) and tinea pedis (athlete’s foot), the last one being the most prevalent fungal infection in developed countries, and the first one accounting for 50% and 90% of all fingernail and toenail infections, respectively [30].

**Table 4:** Minimum Inhibitory Concentration (MIC_100_, µg mL^−1^) of **2**–**11** and **18** against clinical isolates of *Trichophyton* genus.

Strain	Voucher specimen	2	3	4	6	7	8	9	10	11	18	Terb.
*T. rubrum*	C 110	16	16	62	62	31	125	62	31	31	125	0.006
*T. rubrum*	C 135	31	31	62	62	62	125	125	31	31.	125	0.006
*T. rubrum*	C 136	31	31	62	125	62	125	125	62	62	125	0.006
*T. rubrum*	C 137	16	31	31	62	31	125	125	31	62	125	0.006
*T. rubrum*	C 139	16	16	31	62	31	125	62	62	62	125	0.012
*T. rubrum*	C 140	16	62	16	62	31	125	62	62	31	125	0.003
												
*T. mentagroph* *ytes*	C 108	16	125	62	62	31	125	62	62	62	125	0.006
*T. mentagroph* *ytes*	C 364	16	62	31	62	31	250	62	62	62	125	0.006
*T. mentagroph* *ytes*	C 539	31	125	16	62	31	250	62	62	125	125	0.006
*T. mentagroph* *ytes*	C 738	16	62	31	62	31	125	125	62	31	125	0.006
*T. mentagroph* *ytes*	C 943	31	62	16	62	62	250	62	62	62	125	0.006
*T. mentagroph* *ytes*	C 944	31	31	31	62	62	125	62	62	31	125	0.006

C = Mycological Reference Center (Rosario, Argentina), Terb. = Terbinafine.

Results showed that the activity of each compound was similar against all strains, being again compound **2** the most active among the whole series of compounds.

*(d) Relationship between lipophilicity and antifungal behavior*: In order to understand if the antifungal activity of the eugenol derivatives tested here was related to their hydrophilicity, as stated by Sikemma *et al.* [19] and Gill *et al.* [20], or to lipophilicity, as found by Voda *et al.* [21], the *log P* of each eugenol derivative was calculated and compared with the different MIC values. Results showed that there was not a neat correlation between MIC and lipophilicity for any type of fungi tested (Table 1). For example compound **2**, which possesses *log P* = 2.65, has a lower MIC mainly against *C. albicans* and *C. neoformans* than **3** or **4**, which possess the same *log P* (Table 1).

*(e) Mode of action studies*: To determine the mode of action of the most active compound **2** on the integrity of the fungal cell-wall, the Sorbitol Protection Assay was performed [31]. In this assay, MIC determinations were conducted in parallel with and without 0.8 mol L^−1^ sorbitol, an osmotic protectant used for stabilizing fungal protoplasts. It is expected that the MIC of a compound that damages the cell-wall will shift to a much higher value in the presence of the osmotic support [31]. Results showed that MIC of **2** did not vary in the presence of sorbitol after seven days of incubation, for any of the yeasts tested (results not shown), suggesting that **2** would not act by inhibiting the mechanisms controlling cell-wall synthesis or assembly. 

**Figure 2:**
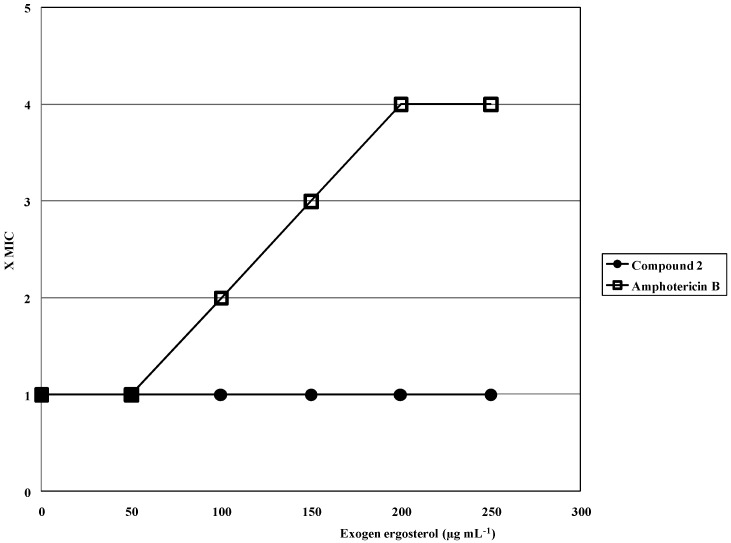
Effect of exogen ergosterol (50–250 µg mL^−1^) on the MIC of both, 6-NO_2_ eugenol (**2**) and amphotericin B against *C. albicans* ATCC 10231. On the “y” axis: 1 = 1× MIC; 2 = 2× MIC; 4 = 4× MIC.

To determine if **2** damages the fungal membrane, the “Ergosterol Effect Assay” was performed.This test detects if a compound acts by binding to the ergosterol of the fungal membrane and is based on offering exogen ergosterol to a compound which, when possessing affinity with it, will rapidly form a complex, thus preventing the complexation with the membrane’s ergosterol. As a consequence, an enhancement of MIC is observed [32,33]. Results showed (Figure 2) that MIC of **2** against *C. albicans ATCC 10231* cells remains unchanged in the presence of different concentrations (50 to 250 µg mL^−1^) of exogenous ergosterol, therefore suggesting that this compound did not act by binding to the membrane. In contrast, a 4-fold increase of MIC was observed for the positive control drug amphotericin B, whose interaction with ergosterol is well-known [34,35].

**Figure 3:**
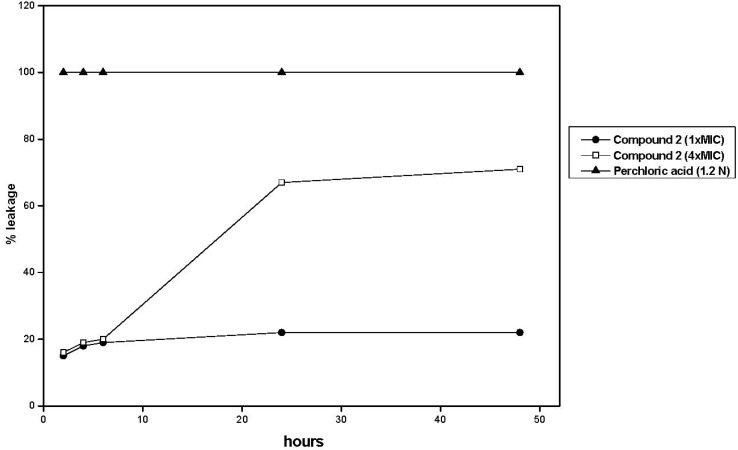
Release of 260-UV absorbing materials from cells of *C. albicans* ATCC 10231incubated (2–48 h) with 1× and 4× MFC of 6-NO_2_ eugenol **2**.

An extra assay, the “Cellular Leakage Assay” was performed to assess if compound 2 produces fungal membrane damage [33]. It is based on the assumption that a disruption of the membrane will cause a release of intracellular components from the fungal cell. Cellular components which absorb at 260 nm represent one class of leakage components, primarily nucleotides of which uracil-containing compounds exhibit the strongest absorbance [33]. Compound 2 (1× MIC and 4× MIC in two separate experiments) was added to cell suspensions of *C. albicans* and the samples were examined at several time intervals (2, 4, 6, 24 and 48 h). Results showed (Figure 3) that 1× MIC of 2 produced increases of 15, 18, 19, 22 and 22% on OD_260_ at 2, 4, 6, 24 and 48 h, relative to perchloric acid that is considered to produce 100% leakage (*p* < 0.001). In turn, 4× MIC of 2 produced enhancements of leakage of 16, 19, 20, 67 and 71% in the same intervals.

Based on the above experiments, it can be stated that **2** does not produce alterations to the fungal cell-wall but rather disrupts fungal membranes at 1× and 4× MIC, which is not due to the binding to membrane’s ergosterol.

## 3. Experimental

### 3.1. General

IR spectra were obtained in a Thermo Scientific Nicolet Impact 6700 FT-IR spectrometer using KBr pellets or as thin films and frequencies are reported in cm^−1^. ^1^H- and ^13^C-NMR (DEPT 135 and DEPT 90) were performed on a Bruker Avance 400 Digital NMR spectrometer, operating at 400.1 MHz for ^1^H and 100.6 MHz for ^13^C; some spectra were recorded in CDCl_3_ solutions and are referenced to the residual peaks of CHCl_3_, *δ* = 7.26 ppm and *δ* = 77.0 ppm for ^1^H and ^13^C, respectively, other spectra were recorded in CD_3_COCD_3_ solutions and are referenced to the residual peaks of CH_3_COCH_3_, *δ* = 2.04 ppm and *δ* = 29.8, *δ* = 206.0 ppm for ^1^H and ^13^C, respectively. Chemical shifts are reported in *δ* ppm and coupling constants (*J*) are given in Hz. Low resolution mass spectra were recorded on a Shimadzu QP-2000 spectrometer at 70 eV ionising voltage and are given as *m/z* (% rel. int.). High resolution mass spectra were recorded on a LTQ Orbitrap XL spectrometer by applying a voltage of 1.8 kV in the positive and 1.9 kV in the negative ionization mode. The spectra were recorded using full scan mode, covering a mass range from *m/z* 100–1,300. The resolution was set to 50,000 and the maximum loading time for the ICR cell was set to 250 ms. Silica gel (Merck 200–300 mesh) was used for CC and silica gel plates GF-254 for TLC. TLC spots were detected by UV light and by heating after spraying with 25% H_2_SO_4_ in H_2_O. UV spectra were recorded in a Beckman DU-640 spectrophotometer (Missouri, Texas, USA).

*2-Methoxy-4-allyl-5-nitrophenol* (**3**). A solution containing 2-methoxy**-**4-allyl-5-nitrophenyl acetate (0.2041 g, 0.8 mmol) in methanol (20 mL) and K_2_CO_3_ (20 mg, 0.14 mmol) was stirred overnight at r.t. Then, 0.1 M HCl was added (until pH 2) and the organic phase was extracted with CH_2_Cl_2_ (3 × 15 mL), washed with water, dried over Na_2_SO_4_, filtered and evaporated. Subsequently, the mixture was purified by column chromatography (CC) eluting with petroleum ether/EtOAc mixtures of increasing polarity to give compound **3** (0.1438 g, 85%) as oil. IR (cm^−^^1^): 3,386 (OH); 1,522 (NO); 1,328 (NO); 1,276 (CO); 1,655 (C=C). HRMS (EI): *m/z* calcd. for C_10_H_11_NO_4_ [M+1]^+^ 210.0688, found 210.0692. ^1^H-NMR: 7.64 (s, 1H, H-3); 7.26 (s, 1H, H-6); 5.96 (m, 1H, H-2′); 5.67 (s, 1H, OH); 5.10 (m, 2H, H-3′); 3.98 (s, 3H, OCH_3_); 3.68 (dd, 2H, *J* = 6.4 Hz; and 1.3 Hz H-1′) ^13^C-NMR: 150.3 (C-2); 143.9 (C-1); 141.9 (C-5); 135.6 (C-2′); 129.0 (C-4); 116.7 (C-3′); 112.5 (C-3); 111.6 (C-6); 56.3 (OCH_3_); 37.4 (C-1′).

*2-Methoxy-3-nitro-4-allylphenol* (**4**). A solution containing 2-methoxy**-**3-nitro-4-allylphenyl acetate (0.205 g, 0.8 mmol) in methanol (20 mL) and K_2_CO_3_ (40 mg, 0.28 mmol) was stirred overnight at r.t. Then, 0.1 M HCl was added to the mixture (until pH 2) and the organic phase was extracted with CH_2_Cl_2_ (3 × 15 mL), washed with water, dried over Na_2_SO_4_, filtered and evaporated. Subsequently, the mixture was chromatographed by CC eluting with petroleum ether/EtOAc mixtures of increasing polarity to give a quantitative yield (0.17 g) of a yellow oil corresponding to the desired product **4**. IR (cm^−^^1^): 3,448 (OH); 1,531 (N-O); 1,504 (C=C); 1,372 (N-O); 1,280 (C=O); 830 (C-H). HRMS (EI): *m/z* calcd. for C_10_H_11_NO_4_ [M+1]^+^ 210.0688, found 210.0690. ^1^H-NMR: 7.03 (d, 1H, *J* = 8.7 Hz, H-5); 6.94 (d, 1H, *J* = 8.5 Hz; H-6); 5.84 (m, 1H, H-2′); 5.65 (s, 1H, OH); 5.09 (m, 2H, H-3′); 3.90 (s, 3H, OCH_3_); 3.03 (d, 2H, *J* = 6.5 Hz, H-1′) ^13^C-NMR: 148.0 (C-2); 138.9 (C-1 and C-3); 134.8 (C-2′); 126.1 (C-5); 124.2 (C-4); 118.1 (C-6); 117.2 (C-3′); 62.7 (OCH_3_); 34.9 (C-1′).

*2-Methoxy-3,5-dinitro-4-allylphenol* (**5**). Compound **14** (0.2150 g, 0.10 mmol) dissolved in CH_2_Cl_2_ (15 mL) was added to a mixture containing KHSO4 (0.705 g, 33 mmol), NaNO_3_ (0.525 g, 35.3 mmol) and wet silica 50% W/W (0.549 g). The mixture was stirred 3 days at r.t., then filtered, and the solid was washed with CH_2_Cl_2_ and the solvent was evaporated under vacuum to give a reddish oil. Pure compound **5** (0.1182 g, 35%) was as a red oil obtained by repeated CC eluted with petroleum ether/EtOAc mixtures of increasing polarity. IR (cm^−^^1^): 3,450 (OH); 1,540 (N-O); 1,500 (C=C); 1,365 (N-O); 1,299 (C=O); 810 (C-H). HRMS (EI): *m/z* calcd. for C_10_H_11_NO_4_ [M+1]^+^ 255.0539, found 255.05942. ^1^H-NMR: 9.64 (s, 1H, OH); 6.90 (s, 1H, H-6); 5.85 (m, 1H, H-2′); 5.17 (ddt, 2H, *J* = 18.5 Hz, 10.0 and 1.3 Hz, H-3′); 4.00 (s, 3H, OCH_3_); 3.35 (d, 2H, *J* = 6.5 Hz, H-1′) ^13^C-NMR: 150.8 (C-2); 143.8 (C-1); 133.6 (C-5); 125.5 (C-2′); 118.6 (C-4); 115.7 (C-3′); 117.2 (C-3); 57.0 (OCH_3_); 35.3 (C-1′).

*4-Allyl-1,2-phenyldiacetate* (**9**). To a cold suspension of anhydrous AlCl_3_ (1.12 g, 8.4 mmol) in anhydrous CH_2_Cl_2_ (10 mL), a cold solution of safrole (**1**, 500 mg, 3.1 mmol) in anhydrous CH_2_Cl_2_ (10 mL) was added dropwise under a nitrogen atmosphere and the reaction was stirred for 2 h at −10 °C. Then, the ice bath was removed and cold water (80 mL) was added, maintaining the stirring 24 h. The reaction mixture was poured into a saturated NaHCO_3_ solution and extracted with EtOAc (3 × 100 mL). The organic layer was washed with water, then dried over anhydrous MgSO_4_, filtered, evaporated and re-dissolved in acetone (5 mL). Subsequently, it was adsorbed on a silica gel column and chromatographed eluting with mixtures of petroleum ether/EtOAc of increasing polarity (17.0:3.0→15.0:5.0) to give an oil (0.310 g), which corresponded to a mixture of compounds. This mixture was dissolved in anhydrous CH_2_Cl_2_ (50 mL) and DMAP (3.06 mg) and Ac_2_O (0.40 mL, 4.23 mmol) were added. The reaction mixture was stirred for 2 h at r.t., cooled to 0 °C and KHSO_4_ (10%, 50 mL) were added. Then, the mixture was extracted with EtOAc (3 × 50 mL) washed with water, dried over anhydrous MgSO_4_, filtered, evaporated and re-dissolved in CH_2_Cl_2_ (5 mL). Subsequently, it was adsorbed on silica gel and chromatographed eluting with petroleum ether/EtOAc mixtures of increasing polarity to give **9** as an oil (313 mg, 43.2%); IR (cm^−1^): 2,957 (=C-H); 1,768 (C=O); 1,636 (C=C); 1,371 (CH_3_); 1,232 (C-O); 905 (-CH=CH_2_). MS (*m/z*, %): [M]^+^ 234 (13.0); 192 (54.9); 175 (26.6); 152 (25.4); 150 (100); 133 (21.5); 131 (21.6); 123 (27.7); 116 (24.3); 104 (19.6); 91 (18.7); 77 (15.4). HRMS (EI): *m/z* calcd. for C_10_H_11_NO_4_ [M+1]^+^ 235.0892, found 235.0895. ^1^H-NMR: 7.08 (m, 2H, H-3 and H-6); 7.00 (dd, 1H, *J* = 8.7 Hz and *J* = 1.5 Hz, H-5); 5.93 (ddt, 1H, *J* = 16.8 Hz; 10.1 and 6.8 Hz, H-2′); 5.12 (dd, 1H, *J* = 6.3 Hz and *J* = 1.3 Hz, H-3′a); 5.09 (t, 1H, *J* = 1.3 Hz, H-3′b); 3.38 (d, 2H, *J* = 6.8 Hz, H-1′); 2.28 (s, 6H, CH_3_CO). ^ 13^C-NMR: 168.4 (CH_3_CO); 168.3 (CH_3_CO); 141.8 (C-4); 140.2 (C-2); 138.9 (C-1); 136.4 (C-2′); 126.6 (C-5); 123.3 (C-3); 123.1 (C-6); 116.6 (C-3′); 39.4 (C-1′); 20.6 (2× CH_3_CO).

*4-Allyl-5-nitro-1,2-phenyldiacetate* (**10**). A solution containing compound **7** (0.38 g, 1.92 mmol), DMAP (3.75 mg) and of Ac_2_O (0.36 mL, 3.84 mmol) in anhydrous CH_2_Cl_2_ (50 mL) was stirred 2 h at r.t. Then, the mixture was cooled to 0 °C and a 10% KHSO_4_ solution (50 mL) was added. The organic phase was extracted with EtOAc (3 × 50 mL), washed with water, dried over anhydrous MgSO_4_, filtered, evaporated and re-dissolved in CH_2_Cl_2_ (5 mL). Subsequently, it was adsorbed on silica gel and chromatographed (CC) eluting with petroleum ether/EtOAc mixtures of increasing polarity to afford **10** as a brown solid (0.50 g, 94.3%); m.p. 62.0–63.7 °C. IR (cm^−1^): 3,083 (=C-H); 2,938 (C-H); 1,779 (C=O); 1,639 (C=C); 1,527 (C=C); 1,370 (CH_3_); 1,272 (C-O-C). MS (*m/z*, %): [M]^+^ 279 (<1%); 237 (18.4); 220 (25.2); 195 (48.1); 179 (12.9); 178 (100); 165 (40.1); 164 (21.8); 161 (25.0); 149 (11.3); 147 (13.3). HRMS (EI): *m/z* calcd. for C_10_H_11_NO_4_ [M+1]^+^ 280.0743, found 280.0748. ^1^H-NMR: 7.87 (s, 1H, H-6); 7.21 (s, 1H, H-3); 5.92 (ddt, 1H, *J* = 17.1 Hz; 10.2 and 6.6 Hz, H-2′); 5.12 (m, 2H, H-3′); 3.67 (d, 2H, *J* = 6.6 Hz, H-1′); 2.30 (s, 6H, CH_3_CO). ^ 13^C-NMR: 167.5 (CH_3_CO); 167.3 (CH_3_CO); 145.7 (C-5); 145.5 (C-2); 140.3 (C-1); 134.2 (C-2′); 134.0 (C-4); 126.2 (C-3); 120.6 (C-3′); 117.9 (C-6); 36.5 (C-1′); 20.4 (CH_3_CO); 20.3 (CH_3_CO).

*4-Allyl-5-nitrobenzene-1,2-diol* (**11**). A solution of **13** (0.30 g, 1.5 mmol) in CH_2_Cl_2_ (7.0 mL) was slowly added to a cold suspension (0 °C) of AlCl_3_ (0.68 g, 5.1 mmol) in CH_2_Cl_2_ (5.0 mL) under nitrogen atmosphere. The resulting mixture was stirred 2 h at −10 °C and cold water (approx. 10 mL) was added to the mixture, which was then stirred for 18 h at r.t. under nitrogen and then poured into brine and extracted with EtOAc (3 × 100 mL). The organic layer was washed with brine and then dried over anhydrous MgSO_4_, filtered, evaporated and re-dissolved in acetone (5 mL). Then, it was adsorbed on silica gel and chromatographed (CC) eluting with mixtures of petroleum ether/EtOAc of increasing polarity (17.0:3.0→15.0:5.0) to give **11** as an oil (0.16 g, 57.4%). IR (cm^−1^): 3,311 (O-H); 2,907 (C-H); 1,598 (C=C); 1,526 (NO_2_); 1,495 (C=C); 1,429 (-CH_2_); 1,326 (N=O); 1,275 (C-O); 1,045 (-C-OH); 809 (-C-H). ^1^H-NMR: 8.99 (bs, 2H, OH); 7.57 (s, 1H, H-6); 6.84 (s, 1H, H-3); 5.95 (ddt, 1H, 1H, *J* = 17.0 Hz, 10.3 and 6.5 Hz, H-2′, H-2′); 5.03 (m, 2H, H-3′); 3.61 (d, 2H, *J* = 6.4 Hz, H-1′); ^13^C-NMR: 151.3 (C-2); 144.4 (C-1); 141.4 (C-5); 137.1 (C-2′); 129.7 (C-4); 118.4 (C-3′); 116.3 (C-3); 113.0 (C-6); 37.6 (C-1′).

*4-Allyl-5-nitro-1,2-methylenedioxy benzene* (**13**). To a cold (−5 °C) solution of safrole (**12**, 2.0 g, 12.3 mmol) in acetic acid (8 mL), a mixture of nitric and sulfuric acids (10:1 ratio, 2.5 mL) was slowly added dropwise at −5 °C. and then stirred 4 h at −10 °C. Water (10 mL) was added and the mixture was extracted with EtOAc (3 × 50 mL). The aqueous layer was discarded and the organic layer was neutralized with a saturated solution of NaHCO_3_. The organic layer was dried over anhydrous MgSO_4_, filtered, evaporated and re-dissolved in CH2Cl2 (5 mL). Subsequently, it was adsorbed on silica gel and chromatographed (CC) eluting with mixtures of petroleum ether/EtOAc of increasing polarity (19.8:0.2→17.8:2.2) to give **13** (1.86 g, 75.0%) as a viscous oil. IR (cm^−1^): 3,081 (=C-H); 2,912 (C-H); 1,616 (C=C); 1,523 (-NO_2_); 1,480 (C=C); 1,421 (-CH_2_); 1,328 (N=O); 1,257 (C-O-C); 927 (-C-O-C-); 817 (-C-H). MS (*m/z*, %): [M+1]^+^ 208 (2.8); [M]^+^ 207 (23.0); 190 (100); 177 (21.0); 176 (17.5); 173 (50.2); 162 (16.9); 160 (23.0); 132 (29.7); 103 (24.9); 102 (51.3). HRMS (EI): *m/z* calcd. for C_10_H_9_NO_4_ [M+1]^+^ 208.0532, found 280.0535. ^1^H-NMR: 7.49 (s, 1H, H-6); 6.76 (s, 1H, H-3); 6.09 (s, 2H, OCH_2_O); 5.95 (ddt, 1H, 1H, *J* = 17.0 Hz, 10.3 and 6.5 Hz, H-2′); 5.10 (m, 2H, H-3′); 3.65 (d, 2H, *J* = 4.0 Hz, H-1′); ^13^C-NMR: 151.7 (C-2); 146.5 (C-5 and C-1); 135.2 (C-2′); 132.2 (C-4); 117.0 (C-3′); 110.4 (C-3); 105.7 (OCH_2_O); 102.7 (C-6); 37.6 (C-1′).

*4-(3-Hydroxypropyl)-5-nitrobenzene-1,2-diol* (**14**). BH_3_·DMS in THF (2.0 M, 0.20 mL) was added dropwise under a nitrogen atmosphere at −10 °C to compound **11** (70 mg, 0.4 mmol) with stirring. Then, the reaction was allowed to reach r.t. and it was maintained 1 h at this temperature. The resulting intermediate was oxidized with a solution of NaBO_3_·4H_2_O (0.1 g, 0.7 mmol) in water (100 mL). The mixture was stirred 2 h at r.t., the organic phase was extracted with ether (4 × 50 mL), washed with water, dried over anhydrous MgSO_4_, filtered, evaporated and re-dissolved in acetone (5 mL). Subsequently, it was subjected to CC eluting with mixtures of petroleum ether/EtOAc to give a yellow solid which upon recrystallization from MeOH/ethyl ether, gave pure **14** (37.5 mg, 49.3%); m.p.: 98.9-99.8 °C. IR (cm^−1^): 3,458 (O-H); 2,921 (=C-H); 1,593 (C=C); 1,532 (NO_2_); 1,457 (CH_2_); 1,385 (CH_3_); 1,331 (N=O); 880 (-C-H). ^1^H-NMR: 9.24 (s, 1H, OH); 8.88 (s, 1H, OH); 7.53 (s, 1H, H-6); 6.85 (s, 1H, H-3); 5.89 (b.s, 1H, OH); 4.39 (t, 2H, *J* = 6.4 Hz, H-3′); 2.88 (m, 2H, H-1′), 1.79 (m, 2H, H-2′). ^13^C-NMR: 151.3 (C-2); 144.2 (C-1); 141.5 (C-5); 132.2 (C-4); 118.5 (C-3); 112.9 (C-6); 61.8 (C-3′); 34.4 (C-2′); 30.1 (C-1′).

*4*-*[3-(Acetyloxy)propyl]-1,2-phenyl diacetate* (**15**). To compound **9** (250 mg, 1.1 mmol) BH_3_·DMS in THF (2.0 M, 0.25 mL) was added slowly under a nitrogen atmosphere, over a period of 15 min, making sure to keep the temperature at −10 °C. Then, the reaction was taken to r.t. and maintained 1h at this temperature. The resulting organoborane intermediate was oxidized with a solution containing NaBO_3_·4H_2_O (0.24 g, 1.6 mmol) in water (100 mL). The mixture was stirred 2 h at r.t. The organic phase was extracted with ether (4 × 50 mL), dried over anhydrous Na_2_SO_4_, filtered, evaporated and re-dissolved in 5 mL of acetone. Subsequently, it was subjected to CC eluting with mixtures of petroleum ether/EtOAc yielding 147.4 mg of a solid which corresponded to a mixture of compounds. The mixture was dissolved in anhydrous CH_2_Cl_2_ (50 mL) and DMAP (1.02 mg) and Ac_2_O (0.40 mL, 4.23 mmol) were added. The reaction was stirred 2 h at r.t. and then cooled at 0 °C. KHSO_4_ (10%, 50 mL) was added, and the mixture was extracted with EtOAc (3 × 50 mL), washed with water, dried over MgSO_4_, filtered, evaporated and re-dissolved in CH2Cl2 (5 mL). Subsequently, it was adsorbed on silica gel and chromatographed by CC eluting with petroleum ether/EtOAc mixtures of increasing polarity to give a colorless oil which corresponded to the desired product **15** (139 mg, 44.1%). IR (cm^−1^): 2,925 (=C-H); 1,731 (C=O); 1,561 (C=C); 1,429 (-CH_2_); 1,214 (C-O-Ar); 1,076 (AcO); 896 (C-H). MS (*m/z*, %): [M]^+^ 294 (25.7); 282 (18.4); 281 (64.2); 267 (11.5); 222 (18.7); 221 (80.6); 207 (33.6); 147 (78.6); 73 (100). HRMS (EI): *m/z* calcd. for C_10_H_9_NO_4_ [M+1]^+^ 295.1103, found 295.1106. ^1^H-NMR: 7.08 (m, 2H, H-5 and H-6); 7.01 (s, 1H, H-3); 4.10 (t, 2H, *J* = 6.5 Hz, H-3′); 2.68 (t, 2H, *J* = 7.8 Hz, H-2′); 2.26 (s, 6H, CH_3_CO); 2.05 (s, 3H, CH_3_CO); 1.98 (m, 2H, H-2′). ^13^C-NMR: 171.1 (CH_3_CO); 168.4 (CH_3_CO); 168.3 (CH_3_CO); 141.9 (C-2); 140.2 (C-1); 140.1 (C-4); 126.5 (C-5); 123.2 (C-3); 123.1 (C-6); 63.6 (C-3′); 31.6 (C-2′); 29.9 (C-1′); 20.9 (CH3); 20.6 (2× CH_3_).

*3-(3′,4′-Methylenedioxy)phenylpropanol* (**16**). Compound **12** (1.0 g, 6.2 mmol), was hydroborated with a 2.0 M solution of BH_3_·DMS/THF (0.67 mL) added dropwise during 15 min under a nitrogen atmosphere at −10 °C. Then, the mixture was stirred 1 h at r.t. The resulting organoborane was oxidized with sodium perborate (0.95 g, 6.2 mmol) in water (100 mL). The mixture was stirred 2 h at r.t. Then, it was extracted with ethyl ether (4 × 50 mL) and the layers were separated. The organic layer was dried over anhydrous MgSO_4_, filtered, evaporated and re-dissolved in CH_2_Cl_2_ (5 mL). It was adsorbed on a silica gel column and chromatographed eluting with mixtures of petroleum ether/EtOAc of increasing polarity (18.8:1.2→17.6:2.4) to give 0.66 g (59.4%) of compound **16** as a viscous oil; **IR** (cm^−1^): 3,330 (O-H); 2,909 (C-H); 1,495 (C=C); 1,439 (-CH_2_); 1,245 (C-O-C); 1,039 (-C-OH); 932 (C-O-C); 811 (-C-H). MS (*m/z*, %): [M+1]^+^ 181 (6.2); [M]^+^ 180 (51.6); 136 (51.2); 135 (100); 119 (5.4); 106 (9.5); 105 (7.8); 104 (5.1); 91 (10.5. HRMS (EI): *m/z* calcd. for C_10_H_12_O_3_ [M+1]^+^ 181.0786, found 181.0790. ^1^H-NMR: 6.73 (d, 1H, *J* = 7.6 Hz, H-6); 6.69 (d, 1H, *J* = 1.4 Hz, H-3); 6.64 (dd, 1H, *J* = 1.4 and *J* = 7.6 Hz, H-5); 5.91 (s, 2H, OCH_2_O); 3.65 (t, 2H, *J* = 6.4 Hz, H-3′); 2.62 (t, 2H, *J* = 7.4 Hz, H-1′); 1.84 (dt, 2H, *J* = 6.4 and *J* = 15.2 Hz, H-2′); 1.56 (bs, 1H, OH); ^13^C-NMR: 147.5 (C-2); 145.6 (C-1); 135.6 (C-4); 121.1 (C-5); 108.8 (C-6); 108.1 (C-3); 100.7 (OCH_2_O); 62.1 (C-3′); 34.4 (C-1′); 31.7 (C-2′).

*3-(2′-Nitro-4′,5′-methylenedioxy)phenyl propanol* (**17**). A 2.0 M solution of BH_3_·DMS/THF (0.27 mL) was added dropwise over 15 min at −10 °C to compound **13** (0.30 g, 1.5 mmol) under a nitrogen atmosphere, and the mixture was stirred 1 h at r.t. The resultant organoborane was oxidized with sodium perborate (0.28 g, 1.5 mmol) in water (100 mL) and then the mixture was stirred 2 h at r.t. Then, it was extracted with ethyl ether (4 × 50 mL) and the layers were separated. The organic layer was dried over MgSO_4_, filtered, evaporated and re-dissolved in CH_2_Cl_2_ (5 mL). It was adsorbed on silica gel, and chromatographed eluting with mixtures of petroleum ether/EtOAc of increasing polarity (16.0:4.0→14.0:6.0) to give compound **17** (0.17 g, 53.1%) as a yellow solid; m.p. (85.9–87.9 °C); IR (cm^−1^): 3,211 (O-H); 2,907 (C-H); 1,613 (C=C); 1,521 (NO_2_); 1,419 (-CH_2_); 1,337 (N=O); 1,260 (C-O-C); 1,045 (-C-OH); 922 (C-O-C); 825 (-C-H). HRMS (EI): *m/z* calcd. for C_10_H_11_NO_5_ [M+1]^+^ 226.0637, found 226.0639. ^1^H-NMR: 7.46 (s, 1H, H-6); 6.76 (s, 1H, H-3); 6.08 (s, 2H, OCH_2_O); 3.71 (t, 2H, *J* = 6.2 Hz, H-3′); 2.96 (dd, 2H, *J* = 6.4 and *J* = 8.6 Hz, H-1′); 1.90 (m, 2H, H-2′) 1.50 (bs, 1H, OH); ^13^C-NMR: 151.7 (C-2); 146.3 (C-1); 142.8 (C-5); 134.4 (C-4); 110.6 (C-3); 105.7 (C-6); 102.7 (OCH_2_O); 62.0 (C-3′); 33.4 (C-2′); 30.1 (C-1′).

*3-(2′-Nitro-4′,5′-methylenedioxy)phenyl propyl acetate* (**18**). To a solution of **17** (97.8 mg, 0.43 mmol) in dry CH_2_Cl_2_ (30 mL), DMAP (0.98 mg) and Ac_2_O (40.7 μL, 0.43 mmol) were added and the mixture was stirred 2 h at r.t. A solution of 10% KHSO_4_ (approx. 50 mL) was then added to this mixture. The aqueous layer was discarded and the organic layer was taken to neutrality with a saturated solution of NaHCO_3_ and water. It was dried over MgSO_4_, filtered, evaporated and re-dissolved in CH2Cl2 (5 mL), then chromatographed (CC) eluting with petroleum ether/EtOAc mixtures of increasing polarity (19.8:0.2→19.0:1.0) to give **18** as an oil (110.4 mg, 95.1%). IR (cm^−1^): 2,778 (C-H); 1,735 (C=O); 1,619 (C=C); 1,516 (NO_2_); 1,425 (-CH_2_); 1,379 (CH_3_); 1,330 (N=O); 1,260 (C-O-C); 1,255 (C-O-C); 928 (C-O-C); 817 (-C-H). MS (*m/z*, %): [M]^+^ 267 (<1%); 208 (16.0); 191 (13.6); 190 (100); 189 (14.5); 178 (23.1); 173 (9.2); 163 (19.9); 148 (13.7); 136 (13.3); 135 (13.1); 132 (15.7); 104 (9.9); 77 (12.2). HRMS (EI): *m/z* calcd. for C_12_H_13_NO_6_ [M+1]^+^ 268.0743, found 268.0747. ^1^H-NMR: 7.43 (s, 1H, H-6); 6.69 (s, 1H, H-3); 6.05 (s, 2H, OCH_2_O); 4.07 (t, 2H, *J* = 6.3 Hz, H-3′); 2.89 (m, 2H, H-1′); 2.03 (s, 3H, CH_3_); 1.93 (m, 2H, H-2′). ^13^C-NMR: 170.4 (CH_3_CO); 151.6 (C-2); 146.3 (C-1); 142.6 (C-5); 133.5 (C-4); 110.6 (C-3); 105.6 (OCH_2_O); 102.7 (C-6); 63.4 (C-3′); 30.5 (C-2′); 29.3 (C-1′); 20.8 (CH_3_CO).

*4-(3-Acetoxypropyl)-5-nitro-1,2-phenyl diacetate* (**19**). DMAP (3.75 mg) and Ac_2_O (0.36 mL, 3.84 mmol) were added to a solution of **14** (0.38 g, 1.92 mmol) in dry CH_2_Cl_2_ (60 mL) and the mixture was stirred 2 h at r.t. A solution of 10% KHSO_4_ (approx. 50 mL) was then added to this mixture. The aqueous layer was discarded and the organic layer was washed to neutrality with a saturated solution of NaHCO_3_ and water. Then, it was dried over MgSO_4_, filtered, evaporated and re-dissolved in CH_2_Cl_2_ (5 mL). Subsequently, it was adsorbed on a silica gel column and chromatographed with petroleum ether/EtOAc mixtures of increasing polarity (19.8:0.2→16.4:3.6) to give **19**, (0.50 mg, 94.3%) as a yellow solid; m.p. (62.0–63.7 °C); IR (cm^−1^): 3,083 (=C-H); 2,938 (C-H); 1,779 (C=O); 1,639 (C=C); 1,527 (C=C); 1,370 (CH_3_); 1,272 (C-O-C). MS (*m/z*, %): [M]^+^ 337 (<1%) 237 (18.4); 220 (25.2); 195 (48.1); 179 (12.9); 178 (100); 165 (40.1); 164 (21.8); 161 (25.0); 149 (11.3); 147 (13.3). HRMS (EI): *m/z* calcd. for C_16_H_19_NO_7_ [M+1]^+^ 338.1162, found 338.1166. ^1^H-NMR: 7.87 (s, 1H, H-6); 7.21 (s, 1H, H-3); 5.92 (ddt, 1H, *J* = 17.1 Hz, 10.2 and 6.6 Hz, H-2′); 5.12 (m, 2H, H-3′); 3.67 (d, 2H, *J* = 6.6 Hz, H-1′); 2.30 (s, 6H, CH_3_). ^13^C-NMR: 167.4 (2× CH_3_CO); 145.7 (C-5); 145.5 (C-2); 140.3 (C-1); 134.2 (C-2′); 134.0 (C-4); 126.2 (C-3); 120.6 (C-3′); 117.9 (C-6); 36.5 (C-1′); 20.4 (2× CH_3_CO).

### 3.2. Antifungal Susceptibility Testing

#### 3.2.1. Microorganisms and Media

For the antifungal evaluation, standardized strains from the American Type Culture Collection (ATCC, Rockville, MD, USA), and the Center of Reference in Mycology (CEREMIC, C, Facultad de Ciencias Bioquímicas y Farmacéuticas, Rosario, Argentina) were used in a first instance of screening: *C. albicans* ATCC 10231, *S. cerevisiae* ATCC 9763, *C. neoformans* ATCC 32264, A*spergillus flavus* ATCC 9170, *A. fumigatus* ATTC 26934, *A. niger* ATCC 9029, *Trichophyton rubrum* C 113, *T. mentagrophytes* ATCC 9972, and *M. gypseum* C 115.

Then, active compounds were tested against clinical isolates from CEREMIC and the Malbrán Institute [M, Buenos Aires, Argentina). The isolates included 12 strains of *Candida* spp. (five of them *C. albicans* and seven *Candida* non-*albicans*); nine strains of *C. neoformans*; six strains of *T. rubrum* andsix of *T. mentagrophytes.* The voucher specimen numberx of each isolate are presented in Table 2, Table 3 and Table 4. Strains were grown on Sabouraud-chloramphenicol agar slants for 48 h at 30 °C, maintained on slopes of Sabouraud-dextrose agar (SDA, Oxoid) and sub-cultured every 15 days to prevent pleomorphic transformations. Inocula of cell or spore suspensions were obtained according to reported procedures and adjusted to 1–5 × 10^3^ cells/spores with colony forming units (CFU) mL^−1^ [27,28].

#### 3.2.2. Determination of MICs and MFCs

Minimum Inhibitory Concentration (MIC) of each compound was determined by using broth microdilution techniques according to the guidelines of the CLSI for yeasts (M27-A3) and for filamentous fungi (M 38 A2) [16,17]. MIC values were determined in RPMI-1640 (Sigma, St. Louis, MO, USA) buffered to pH 7.0 with MOPS. Microtiter trays were incubated at 35 °C for yeasts and hialohyphomycetes and at 28–30 °C for dermatophyte strains in a moist, dark chamber, and MICs were visually recorded at 48 h for yeasts, and at a time according to the control fungus growth, for the rest of fungi.

For the assay, stock solutions of pure compounds were diluted two-fold with RPMI from 250–0.98 µg mL^−1^ (final volume = 100 µL) and a final DMSO concentration ≤1%. A volume of 100 µL of inoculum suspension was added to each well with the exception of the sterility control where sterile water was added to the well instead. Ketoconazole, terbinafine, amphotericin B and itraconazole were used as positive controls.

Endpoints were defined as the lowest concentration of drug resulting in total inhibition (MIC_100_) of visual growth compared to the growth in the control wells containing no antifungal. MIC_80_ and MIC_50_ were defined as the lowest concentration of a compound that showed 80% or 50% reduction of the growth control respectively (culture media with the microorganism but without the addition of any compound) and was determined spectrophotometrically with the aid of a VERSA Max microplate reader (Molecular Devices, Sunnyvale, CA, USA). 

The minimum fungicidal concentration (MFC) of each compound against each isolate was also determined as follows: after determining the MIC, an aliquot of sample (5 µL) was withdrawn from each clear well of the microtiter tray and plated onto a 150-mm RPMI-1640 agar plate buffered with MOPS (Remel, Lenexa, KS, USA). Inoculated plates were incubated at 30 °C, and MFCs were recorded after 48 h. The MFC was defined as the lowest concentration of each compound that resulted in total inhibition of visible growth.

#### 3.2.3. Determination of MICs and MFCs

*(a) Sorbitol protection assay*: MIC values were determined using *C. albicans* ATCC 10231 and *C. neoformans* ATCC 32264, by the standard broth microdilution procedure described above. Duplicate plates were prepared: one of them containing two-fold dilutions of **2** from 250 to 0.98 µg mL^−1^ and the other one, containing **2** at the same concentrations plus 0.8 mol L^−1^ sorbitol, in each well, as osmotic support. MICs were read at 2 and 7 days [31]. 

*(b) Ergosterol Effect Assay*: MIC of **2** against *C. albicans* (ATCC 10231) was determined following the guidelines of CLSI as explained above, in the absence and in the presence of different (50–250 µg mL^−1^) concentrations of ergosterol (SIGMA Chemical Co.) added to the assay medium, in different lines of the same microplate [33]. Amphotericin B was used as a control drug. MIC was read at 24 h according to the control fungus growth.

*(c) Cellular Leakage Assay*: Cells of *C. albicans* ATCC 10231 cultured by shaking at 30 °C to early stationary phase (18 h growth), were washed with MOPS and re-suspended in MOPS to prepare the inoculums [32,33]. Eppendorfs (final volume 500 µL) containing inocula (5 × 10^4^ cells mL^−1^) and compound **2**, at 1×, 4× MIC were left from 2 to 48 h. At 2, 4, 6, 24 and 48 h, eppendorfs were centrifuged (5 min at 3,000 rpm) and the supernatants (200 µL) were drawn on the wells of a 96-wells-microplate and thoroughly mixed. The extractable 260 nm-absorbing materials were determined by duplicate in a Beckman Coulter DTX 880 Multimode Detector, considering 100% release the absorbance produced by cells treated with 1.2 mol L^−1^ HClO_4_ at 100 °C, 30 min [32,33]. Results were the media of both measures.

#### 3.2.4. Statistical Analysis

Data were statistical analyzed by the Student’s test. A *p* < 0.05 was considered significant.

## 4. Conclusions

A series of twenty-one phenylpropanoids including eugenol, safrole and synthetic analogues, were evaluated for antifungal properties in a first instance of screening with CLSI standardized non-targeted assays against a panel of human opportunistic pathogenic fungi. Based on MIC results, some structure-activity relationships could be established. All active compounds were tested in a second panel of clinical isolates of *albicans* and non-*albicans Candida* strains, *Cryptococcus neoformans* and dermatophytes. The eugenol derivative 4-allyl-2-OMe-5-NO_2_-phenol (**2**) possesses a high activity in these second panels, and therefore it was submitted to targeted assays to gain insight into its mode of action. Results showed that the antifungal activity of **2** was not reversed in the presence of an osmotic support such as sorbitol, suggesting that it does not act by inhibiting the fungal cell wall synthesis or assembly. On the other hand, **2** did not show to bind to ergosterol up to 250 µg mL^−1^ in the Ergosterol Effect Assay, while a 22% of fungal membrane damage at concentrations = 1× MIC and 71% at 4× MIC, were observed at 48 h in the Cellular Leakage Assay. 

Regarding the influence of compounds’ solubility on the antifungal behavior, the comparison of *log P* and MIC for each compound revealed that the antifungal activity of the eugenol analogues studied here, would not to be related to lipophilicity.
